# The impact of a decision tree–based BOPPPS blended teaching model on cognitive performance in clinical epidemiology

**DOI:** 10.3389/fpubh.2026.1854470

**Published:** 2026-05-25

**Authors:** Junqiang Wang, Ling Zhao, Fengchen Gao, Wenxiu Zhao, Zexing Yang, Ying Chen, Limei He

**Affiliations:** 1 School of Public Health, Kunming Medical University, Kunming, Yunnan, China; 2 Department of Endocrinology, The Second Affiliated Hospital of Kunming Medical University, Kunming, Yunnan, China; 3 Department of Reproductive Genetics, The First Affiliated Hospital of Kunming Medical University, Kunming, Yunnan, China

**Keywords:** blended teaching, BOPPPS model, clinical epidemiology, cognitive performance, decision tree analysis

## Abstract

**Background:**

The BOPPPS (Bridge-in, Objectives, Pre-assessment, Participatory learning, Post-assessment, and Summary) blended teaching model has been increasingly applied in medical education; however, evidence regarding its impact on cognitive performance and the underlying influencing factors remains limited. We hypothesized that the blended teaching model based on BOPPPS can effectively enhance students’ cognitive performance, and conduct research to investigate this hypothesis.

**Objective:**

This study employed a decision tree model to investigate the impact of the BOPPPS-based blended teaching model on cognitive performance in the *Clinical Epidemiology* course and to identify key influencing factors.

**Methods:**

This study was a cross-sectional observational study designed. Postgraduate students enrolled in the *Clinical Epidemiology* course at Kunming Medical University were recruited as study participants between October 1, 2022 and October 1, 2024. Data were collected through an online questionnaire administered via *Wenjuanxing* (Questionnaire Star). A decision tree model was applied to analyze the determinants of cognitive performance.

**Results:**

A total of 1,938 valid questionnaires were obtained, with 1,749 (90.2%) of students demonstrating strong overall cognitive performance. Decision tree analysis identified the following as major influencing factors: improvement in innovation skills; ability to summarize and synthesize fundamental methods taught in the course; depth of mastery of the knowledge presented; ability to grasp challenging course content; and enhancement of comprehensive problem-solving skills.

**Conclusion:**

The BOPPPS-based blended teaching model exerts a significant positive effect on students’ overall cognitive performance in *Clinical Epidemiology*, which supported our hypothesis. Future teaching practice should place greater emphasis on the effective implementation, rational application, and continuous optimization of this instructional approach. Future research should employ longitudinal or experimental designs to further validate causal relationships and optimize instructional strategies.

## Introduction

Since *Clinical Epidemiology* was first introduced by Professor John Paul at Yale University in 1938, the discipline has gradually evolved—through the persistent efforts of numerous scholars—into an emerging foundational field within clinical medicine. As an interdisciplinary domain integrating clinical medicine and epidemiology, clinical epidemiology plays a pivotal role in cultivating postgraduate students’ cognitive skills and research capabilities, as well as in supporting the conduct of clinical research (1–3).

With the rapid advancement of the economy, science, technology, and the Internet, traditional teaching models have struggled to meet contemporary students’ developmental needs and the goals of talent cultivation. Thus, innovation in teaching approaches has become imperative. At our institution, instruction in the *Clinical Epidemiology* course has been structured around a blended teaching approach underpinned by the BOPPPS model. Originally proposed by Douglas Cole at Columbia University, the BOPPPS model comprises six key components—Bridge-in, Objectives, Pre-assessment, Participatory learning, Post-assessment, and Summary—forming a complete and coherent teaching–learning pathway. Evidence has shown that, compared with traditional approaches, the BOPPPS model is more effective in enhancing students’ knowledge acquisition, skills, and higher-order thinking (4–7).

The blended teaching model combines the strengths of traditional face-to-face instruction with online learning, emphasizing a student-centered philosophy. Leveraging rich online resources and integrating approaches such as case-based learning (CBL) and problem-based learning (PBL), it promotes learning continuity from pre-class preparation to in-class participation and post-class consolidation. This approach fosters students’ ability to solve complex problems, cultivate innovation, and develop higher-order thinking skills (8, 9).

However, research regarding the blended teaching model based on BOPPPS within the context of *Clinical Epidemiology* remains scarce (7, 8, 10). Furthermore, existing studies have predominantly focused on evaluating overall teaching effectiveness using traditional statistical methods, offering limited insights into the analysis of influencing factors and the complex associations among them (11–13). Additionally, few studies have applied machine learning (ML) methods, such as decision tree models, to identify the determinants of students’ cognitive performance following the course. Decision tree analysis is a powerful machine learning (ML) method capable of performing both classification and regression, with a decision-making process that is transparent and easy to interpret. By detecting and exploiting interactions among variables, decision tree models can automatically select variables, identifying those with the greatest impact on the outcome while excluding less relevant predictors. Through iterative selection and partitioning, the model identifies optimal split points, thereby improving both interpretability and predictive accuracy (14–17).

Based on this, we hypothesized that a BOPPPS-based blended teaching model could effectively enhance students’ cognitive performance, and we conducted a study to investigate this. Specifically, we employed a decision tree model to examine the impact of this blended model on cognitive performance in *Clinical Epidemiology* and to identify key influencing factors. Ultimately, our findings aim to provide references for future curriculum development, optimize instructional design, and offer theoretical guidance for improving students’ cognitive competencies.

## Methods

### Study design

This study was a cross-sectional observational study designed to evaluate the impact of a BOPPPS-based blended teaching model on students’ cognitive performance.

### Study setting and duration

The study was conducted at Kunming Medical University between October 1, 2022, and October 1, 2024.

### Participants

Using cluster sampling, participants were recruited from the first-year graduate classes of the *Clinical Epidemiology* program. A total of 1,948 questionnaires were collected. After excluding incomplete or logically inconsistent responses, 1,938 valid questionnaires were ultimately included for analysis. All participants used the same standardized *Clinical Epidemiology* textbook and provided informed consent electronically prior to participation through the *Wenjuanxing* platform. Participation was voluntary, and respondents could withdraw at any time. This study was approved by the Ethics Committee of Kunming Medical University (Approval No. KMMU2022MEC027). All participants provided informed consent.

### Course design and implementation

**Figure 1:**
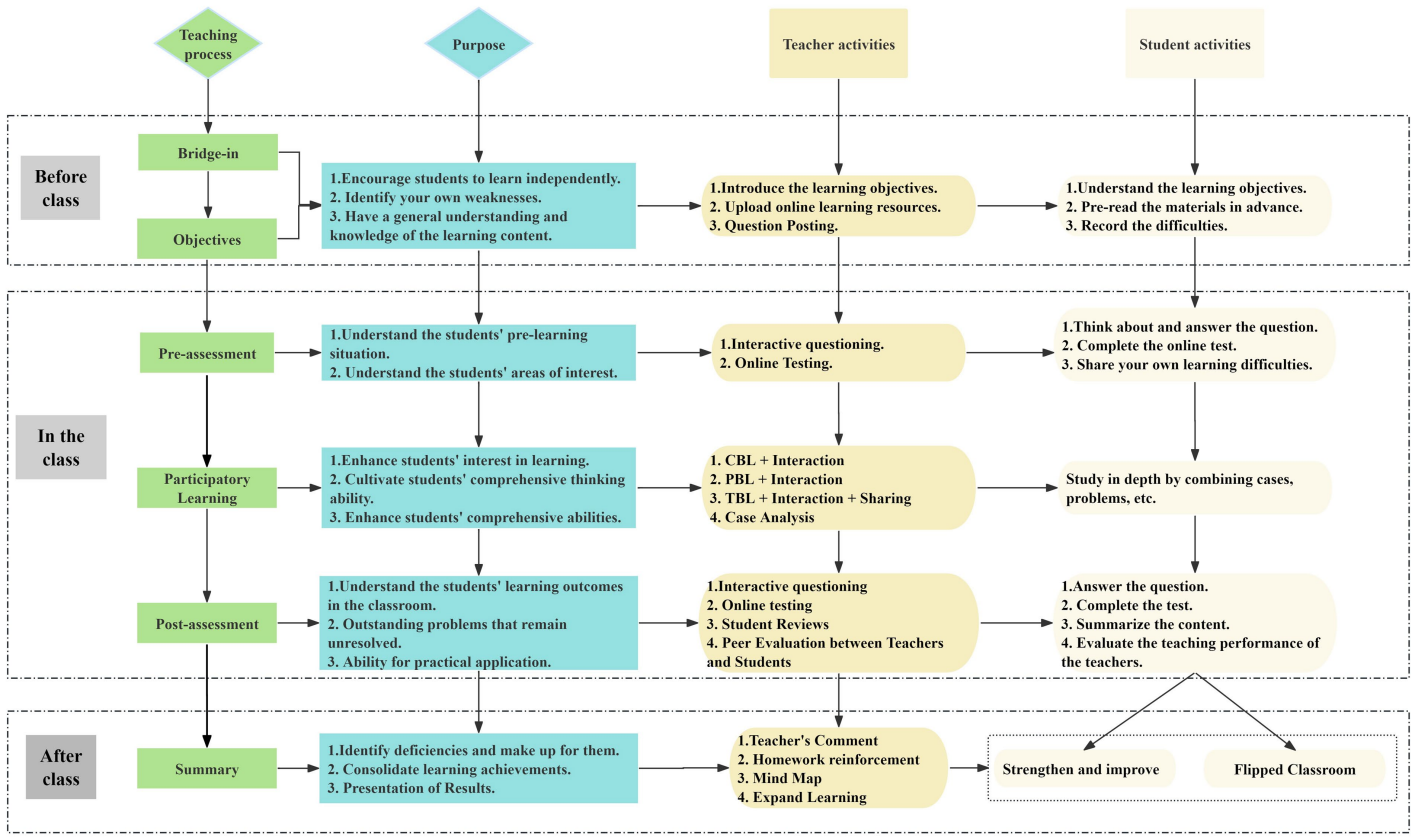
The BOPPPS-based blended teaching model in clinical epidemiology.

The teaching team consisted of six full-time faculty members, each with extensive teaching experience. 5 (83.3%) teachers specialized in epidemiology and health statistics, while 1 (16.7%) teacher specialized in bioengineering. The team included five master’s supervisors and two doctoral supervisors. Instruction was delivered in accordance with the BOPPPS-based blended teaching model (Figure 1).

### Questionnaire survey

Following completion of the *Clinical Epidemiology* course, an online survey was conducted via *Wenjuanxing* (Questionnaire Star). The questionnaire used in our study was developed specifically for this research based on the learning objectives of the *Clinical Epidemiology* course. The questionnaire consisted of six sections: (1) demographic information; (2) knowledge mastery; (3) overall competency performance; (4) overall cognitive performance; (5) evaluation of teaching satisfaction; and (6) evaluation of teaching methods (see Supplementary file 1 for the English version).

The questionnaire demonstrated good reliability and validity, with a Cronbach’s alpha of 0.901 and a Kaiser–Meyer–Olkin (KMO) value of 0.914. In line with established practices in sociology, medical statistics, and economics, categorical variables were coded such that positive/affirmative responses were assigned a score of 1, while negative responses were scored as 0.

To assess the impact of the BOPPPS-based blended teaching model on students’ cognitive performance, the total cognitive performance score (range: 0–6) was categorized into three levels:

- 0–2 points: Weak cognitive performance
- 3–4 points: Moderate cognitive performance
- 5–6 points: Strong cognitive performance

### Data processing and statistical analysis

Data exported from *Wenjuanxing* were cleaned and organized in Microsoft Excel. Incomplete or logically inconsistent questionnaires were excluded. Statistical analyses were conducted using R4.5.1. Continuous variables were expressed as mean ± standard deviation ( $\bar{x}\pm s$ ) or median with interquartile range [*M*(*P*₂₅, *P*₇₅)] and compared using the independent-samples *t* test or the Mann–Whitney *U* test, as appropriate. Categorical variables were expressed as *n* (%) and compared using the chi-square (*χ^2^*) test. Finally, variables with *p* < 0.05 from the univariate analysis were incorporated into the decision tree model, and the CHAID method was employed for decision tree analysis to identify the key influencing factors of cognitive performance. A two-sided *p* value < 0.05 was considered statistically significant.

## Results

### Baseline characteristics

A total of 1,948 questionnaires were collected. After excluding 10 questionnaires with incomplete responses or logical errors, 1,938 valid questionnaires were included in the analysis. Among the participants, 758 (39.11%) were male and 1,180 (60.89%) were female. The age ranged from 20 to 45 years, with a median age of 24 years (IQR: 24–25). Students pursuing a professional degree accounted for 1,685 cases (86.95%), and those pursuing an academic degree accounted for 253 cases (13.05%).

Regarding cognitive performance levels, 1,749(90.2%) students demonstrated strong cognitive performance, 128(6.6%) showed moderate performance, and 61(3.3%) exhibited weak performance. The median (IQR) scores for each dimension were as follows: knowledge mastery: 10 (1), overall competency: 14 (0), cognitive performance: 6 (0), teaching satisfaction: 4 (0), and teaching method evaluation: 6 (1).

### Comparison of different dimensions by demographic characteristics

The study revealed that regarding gender, males scored significantly higher than females in Knowledge Acquisition (*Z* = −4.961), Comprehensive Competency Performance (*Z* = −2.848), and Comprehensive Cognitive Performance (*Z* = −2.212; *p* < 0.05). In terms of household registration, students from urban areas rated the instructional methods significantly higher than those from rural areas (*Z* = −2.283, *p* < 0.05). Regarding degree type, students pursuing academic degrees scored significantly higher in Comprehensive Cognitive Performance (*Z* = −2.223) and evaluation of instructional methods (*Z* = −1.966) compared to those pursuing professional degrees. Notably, students’ subjective attitudes and adaptation status exhibited a more extensive impact: students who were interested in their major, optimistic about its prospects, and adapted to campus life scored significantly higher across multiple dimensions including Knowledge Acquisition, Comprehensive Competency Performance, Comprehensive Cognitive Performance, and teaching satisfaction—than their counterparts (those who were uninterested, pessimistic, or maladapted; *p* < 0.05). Regarding personality, significant differences were observed among different personality types in Knowledge Acquisition (*H* = 9.45, *p* < 0.01) and evaluation of instructional methods (*H* = 21.695, *p* < 0.001). However, ethnicity did not show any statistically significant differences across all measured dimensions (Table 1).

**Table 1:** Comparison of different dimensions across various individual characteristics.

Variable		Knowledge acquisition	Comprehensive competency performance	Comprehensive cognitive performance	Teaching satisfaction	Teaching method evaluation
Gender	Male	10 (9,10)	14 (14,14)	6 (6,6)	4 (4,4)	6 (5,6)
	Female	10 (8,10)	14 (13,14)	6 (6,6)	4 (4,4)	6 (5,6)
*Z*-value		-4.961***	-2.848**	-2.212*	−0.141	−1.182
Ethnicity	Han	10 (9,10)	14 (14,14)	6 (6,6)	4 (4,4)	6 (5,6)
	Minority	10 (8,10)	14 (13,14)	6 (6,6)	4 (4,4)	6 (5,6)
*Z*-value		−0.575	−0.924	−0.22	−0.57	−1.747
Household registration	Urban	10 (9,10)	14 (14,14)	6 (6,6)	4 (4,4)	6 (5,6)
	Rural	10 (8,10)	14 (14,14)	6 (6,6)	4 (4,4)	6 (5,6)
*Z*-value		−1.633	−1.807	−1.455	−1.097	−2.283*
Degree type	Academic	10 (9,10)	14 (13,14)	6 (6,6)	4 (4,4)	6 (5,6)
	Professional	10 (10,9)	14 (14,14)	6 (6,6)	4 (4,4)	6 (5,6)
*Z*-value		−1.03	−1.232	−2.223*	−0.049	−1.966*
Interest in major	Interested	10 (9,10)	14 (14,14)	6 (6,6)	4 (4,4)	6 (5,6)
	Uninterested	8 (6,10)	13 (9,14)	6 (3,6)	4 (4,4)	6 (5,6)
*Z*-value		−6.229***	−6.243***	−7.385***	−2.69**	−1.554
Perception of major prospects	Optimistic	10 (9,10)	14 (14,14)	6 (6,6)	4 (4,4)	6 (5,6)
	Pessimistic	9 (7,10)	14 (11,14)	6 (5,6)	4 (4,4)	6 (5,6)
*Z*-value		−9.627***	−9.761***	−8.37***	−3.901***	−0.207
Campus adaptation	Adapted	10 (9,10)	14 (14,14)	6 (6,6)	4 (4,4)	6 (5,6)
	Not Adapted	7.5 (5,10)	14 (11,14)	6 (4,6)	4 (4,4)	6 (5,6)
*Z*-value		−8.005***	−4.588***	−5.434***	−4.158***	−0.745
Personality	Introverted	10 (8,10)	14 (14,14)	6 (6,6)	4 (4,4)	6 (5,6)
	Extroverted	10 (9,10)	14 (14,14)	6 (6,6)	4 (4,4)	6 (5,6)
	Intermediate	10 (9,10)	14 (14,14)	6 (6,6)	4 (4,4)	6 (5,6)
*H*-value		9.45**	1.092	3.327	3.159	21.695***

***p < 0.001, **p < 0.01, * *p* < 0.05.

### Univariate analysis of cognitive performance levels

Chi-square tests revealed that cognitive performance level was significantly associated (*p* < 0.05) with interest in the major, perceptions of career prospects, and adaptability to the campus environment. Moreover, significant differences (*p* < 0.05) were found in evaluations of the following aspects: knowledge mastery, ability improvement, teaching content, teaching methods, teaching effectiveness, teaching atmosphere, acceptability of teaching content, acceptability of teaching methods, the role of teaching methods in cultivating overall competence, the role of teaching methods in developing clinical professional skills, and the contribution of teaching methods to further academic advancement (Tables 2–5).

**Table 2:** Analysis of differences in cognitive performance by participants’ general characteristics and attitudinal adaptation [*n* (%)].

Variable	Group	*n*	Cognitive performance level			*χ^2^*	*p*
			Low	Moderate	High		
Gender	Male	758	18 (2.4)	43 (5.7)	697 (92.0)	4.401	0.111
	Female	1,180	43 (3.6)	85 (7.2)	1,052 (89.2)		
Ethnicity	Han Chinese	1,557	46 (3.0)	104 (6.7)	1,407 (90.4)	1.018	0.601
	Ethnic Minorities	381	15 (3.9)	24 (6.3)	342 (89.8)		
Residence	Urban	876	24 (2.7)	53 (6.1)	799 (91.2)	1.753	0.416
	Rural	1,062	37 (3.5)	75 (7.1)	950 (89.5)		
Degree type	Academic Degree	253	7 (2.8)	25 (9.9)	221 (87.4)	5.135	0.077
	Professional Degree	1,685	54 (3.2)	103 (6.1)	1,528 (90.7)		
Major liking	Like	1867	52 (2.8)	113 (6.1)	1702 (91.2)	49.515	<0.001
	Dislike	71	9 (12.7)	15 (21.1)	47 (66.2)		
Major prospect view	Good	1,615	39 (2.4)	86 (5.3)	1,490 (92.3)	44.901	<0.001
	Not Good	323	22 (6.8)	42 (13.0)	259 (80.2)		
Campus adaptation	Adapted	1854	51 (2.8)	110 (5.9)	1,693 (91.3)	56.017	<0.001
	Not Adapted	84	10 (11.9)	18 (21.4)	56 (66.7)		
Personality	Introverted	647	20 (3.1)	43 (6.6)	584 (90.3)	4.542	0.338
	Extroverted	268	6 (2.2)	11 (4.1)	251 (93.7)		
	Ambiverted	1,023	35 (3.4)	74 (7.2)	914 (89.3)		

**Table 3:** Analysis of differences in cognitive performance across courses [*n* (%)].

Variable	Group	*n*	Cognitive performance level			*χ^2^*	*p*
			Low	Moderate	High		
Clear learning goals	Yes	1834	42 (2.3)	102 (5.6)	1,690 (92.1)	149.822	<0.001
	No	104	19 (18.3)	26 (25.0)	59 (56.7)		
Define course concepts	Yes	1839	41 (2.2)	103 (5.6)	1,695 (92.2)	166.071	<0.001
	No	99	20 (20.2)	25 (25.3)	54 (54.5)		
Define research scope	Yes	1812	42 (2.3)	106 (5.8)	1,664 (91.8)	92.752	<0.001
	No	126	19 (15.1)	22 (17.5)	85 (67.5)		
Define work tasks	Yes	1818	41 (2.3)	100 (5.5)	1,677 (92.2)	141.447	<0.001
	No	120	20 (16.7)	28 (23.3)	72 (60.0)		
Summarize principles	Yes	1721	30 (1.7)	81 (4.7)	1,610 (93.6)	198.736	<0.001
	No	217	31 (14.3)	47 (21.7)	139 (64.1)		
Summarize methods	Yes	1767	31 (1.8)	90 (5.1)	1,646 (93.2)	211.472	<0.001
	No	171	30 (17.5)	38 (22.2)	103 (60.2)		
Knowledge breadth	Mastered	1,660	26 (1.6)	78 (4.7)	1,556 (93.7)	171.175	<0.001
	Not Mastered	278	35 (12.6)	50 (18.0)	193 (69.4)		
Knowledge depth	Mastered	1,541	18 (1.2)	55 (3.6)	1,468 (95.3)	219.57	<0.001
	Not Mastered	397	43 (10.8)	73 (18.4)	281 (70.8)		
Key points mastery	Mastered	1835	40 (2.2)	111 (6.0)	1,684 (91.8)	127.751	<0.001
	Not Mastered	103	21 (20.4)	17 (16.5)	65 (63.1)		
Difficult points mastery	Mastered	1,542	18 (1.2)	56 (3.6)	1,468 (95.2)	215.531	<0.001
	Not Mastered	396	43 (10.9)	72 (18.2)	281 (71.0)		

**Table 4:** Analysis of differences in cognitive performance across learning abilities [*n* (%)].

Variable	Group	*n*	Cognitive performance level			*χ^2^*	*p*
			Low	Moderate	High		
Efficient learning	Improved	1856	45 (2.4)	106 (5.7)	1705 (91.9)	138.577	<0.001
	Not Improved	82	16 (19.5)	22 (26.8)	44 (53.7)		
Knowledge transfer	Improved	1847	42 (2.3)	103 (5.6)	1702 (92.1)	174.095	<0.001
	Not Improved	91	19 (20.9)	25 (27.5)	47 (51.6)		
Self-directed learning	Improved	1852	42 (2.3)	102 (5.5)	1708 (92.2)	196.765	<0.001
	Not Improved	86	19 (22.1)	26 (30.2)	41 (47.7)		
Problem identification	Improved	1848	36 (1.9)	99 (5.4)	1713 (92.7)	302.094	<0.001
	Not Improved	90	25 (27.8)	29 (32.2)	36 (40.0)		
Problem analysis	Improved	1868	40 (2.1)	102 (5.5)	1726 (92.4)	295.413	<0.001
	Not Improved	70	21 (30.0)	26 (37.1)	23 (32.9)		
Practical problem solving	Improved	1861	39 (2.1)	94 (5.1)	1728 (92.9)	371.097	<0.001
	Not Improved	77	22 (28.6)	34 (44.2)	21 (27.3)		
Complex problem solving	Improved	1783	30 (1.7)	76 (4.3)	1,677 (94.1)	372.999	<0.001
	Not Improved	155	31 (20.0)	52 (33.5)	72 (46.5)		
Literature retrieval	Improved	1875	42 (2.2)	108 (5.8)	1725 (92.0)	232.882	<0.001
	Not Improved	63	19 (30.2)	20 (31.7)	24 (38.1)		
Literature reading	Improved	1854	42 (2.3)	101 (5.4)	1711 (92.3)	212.181	<0.001
	Not Improved	84	19 (22.6)	27 (32.1)	38 (45.2)		
Clinical research skills	Improved	1774	26 (1.5)	78 (4.4)	1,670 (94.1)	378.292	<0.001
	Not Improved	164	35 (21.3)	50 (30.5)	79 (48.2)		
Innovation ability	Improved	1727	20 (1.2)	63 (3.6)	1,644 (95.2)	452.438	<0.001
	Not Improved	211	41 (19.4)	65 (30.8)	105 (49.8)		
Clinical practice skills	Improved	1839	34 (1.8)	96 (5.2)	1709 (92.9)	326.12	<0.001
	Not Improved	99	27 (27.3)	32 (32.3)	40 (40.4)		
Verbal expression	Improved	1821	34 (1.9)	89 (4.9)	1,698 (93.2)	321.846	<0.001
	Not Improved	117	27 (23.1)	39 (33.3)	51 (43.6)		
Teamwork & communication	Improved	1832	36 (2.0)	95 (5.2)	1701 (92.8)	276.058	<0.001
	Not Improved	106	25 (23.6)	33 (31.1)	48 (45.3)		

**Table 5:** Analysis of differences in cognitive performance across teaching evaluations [*n* (%)].

Variable	Group	*n*	Cognitive performance level			*χ^2^*	*p*
			Low	Moderate	High		
Content satisfaction	Satisfied	1926	56 (2.9)	125 (6.5)	1745 (90.6)	67.439	<0.001
	Dissatisfied	12	5 (41.7)	3 (25.0)	4 (33.3)		
Method satisfaction	Satisfied	1908	52 (2.7)	120 (6.3)	1736 (91.0)	95.805	<0.001
	Dissatisfied	30	9 (30.0)	8 (26.7)	13 (43.3)		
Effect satisfaction	Satisfied	1915	53 (2.8)	124 (6.5)	1738 (90.8)	82.749	<0.001
	Dissatisfied	23	8 (34.8)	4 (17.4)	11 (47.8)		
Atmosphere satisfaction	Satisfied	1911	53 (2.8)	123 (6.4)	1735 (90.8)	71.365	<0.001
	Dissatisfied	27	8 (29.6)	5 (18.5)	14 (51.9)		
Content difficulty	Difficult	1,377	42 (3.1)	95 (6.9)	1,240 (90.1)	0.788	0.674
	Not Difficult	561	19 (3.4)	33 (5.9)	509 (90.7)		
Content acceptability	Acceptable	1921	57 (3.0)	124 (6.5)	1740 (90.6)	32.718	<0.001
	Unacceptable	17	4 (23.5)	4 (23.5)	9 (52.9)		
Method acceptability	Acceptable	1919	57 (3.0)	126 (6.6)	1736 (90.5)	21.003	<0.001
	Unacceptable	19	4 (21.1)	2 (10.5)	13 (68.4)		
Quality cultivation	Helpful	1915	54 (2.8)	125 (6.5)	1736 (90.7)	59.457	<0.001
	Not Helpful	23	7 (30.4)	3 (13.0)	13 (56.5)		
Professional skills cult.	Helpful	1905	55 (2.9)	117 (6.1)	1733 (91.0)	66.91	<0.001
	Not Helpful	33	6 (18.2)	11 (33.3)	16 (48.5)		
Adv. studies help	Helpful	1912	57 (3.0)	121 (6.3)	1734 (90.7)	32.105	<0.001
	Not Helpful	26	4 (15.4)	7 (26.9)	15 (57.7)		

### Decision tree model

Using cognitive performance level as the dependent variable, variables with statistical significance (*p* < 0.05) from the univariate analysis were included in a CHAID decision tree model. The model identified three layers, 10 nodes, and six terminal nodes, with five variables influencing cognitive performance.

The first decision node was whether students’ innovation ability had improved. Among students with improved innovation ability, 1,644 (95.2%) students exhibited strong cognitive performance, compared with 105 (49.8%) among those without improvement. The second layer included factors related to knowledge mastery: the ability to summarize and generalize the basic methods of the course, and the ability to master the depth of the taught content.

- For students with improved innovation ability, those who could summarize and generalize the basic methods of the course had a strong cognitive performance rate of 96.4%, compared with 77.6% for those who could not.
- For students without improved innovation ability, those who could master the depth of the taught content had a strong cognitive performance rate of 70.2%, compared with 33.3% for those who could not.

**Figure 2:**
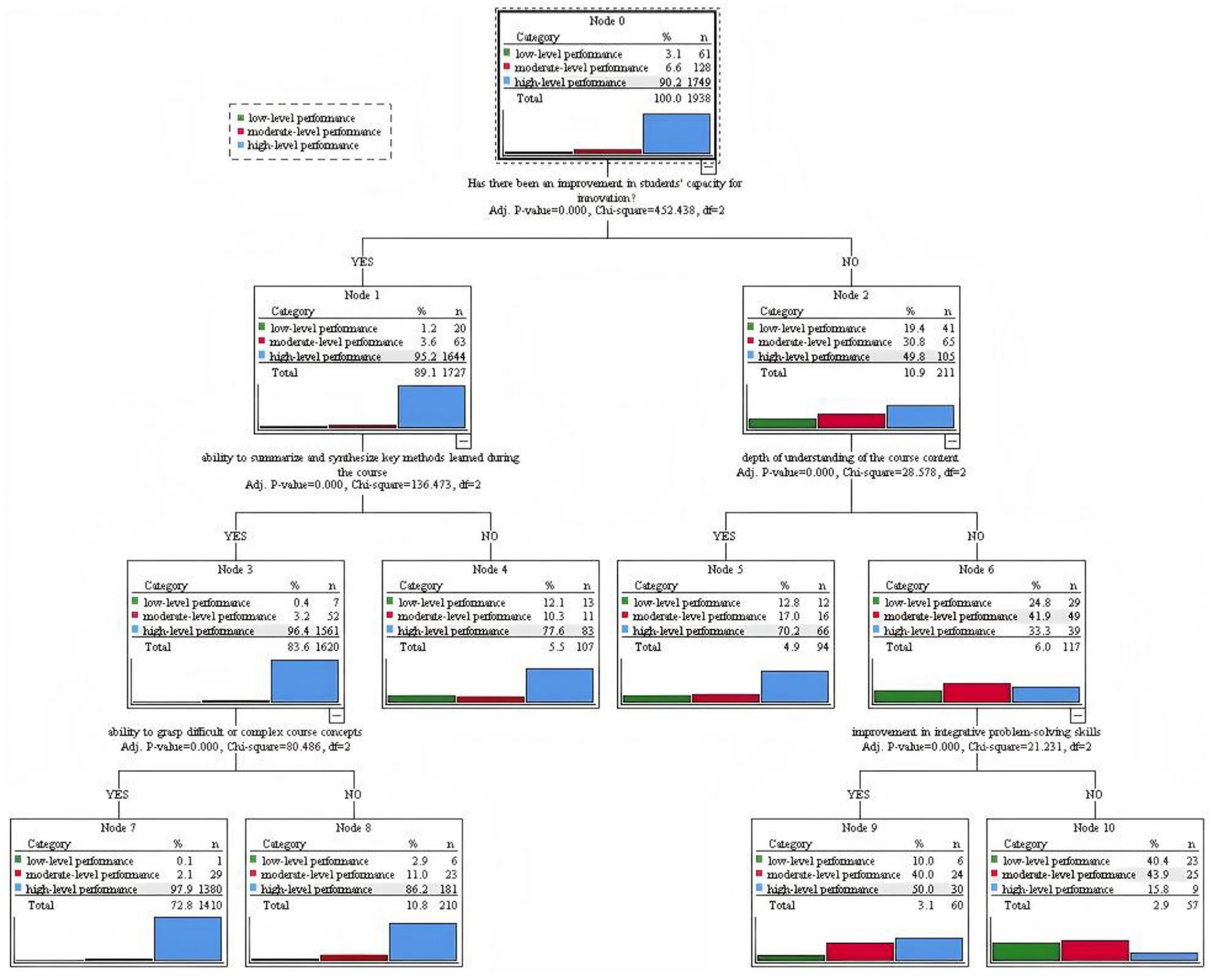
Decision tree analysis of factors influencing students’ cognitive performance.

In the third layer, the selected factors were the ability to master the difficult points of the taught content (for those who could summarize and generalize course methods) and comprehensive problem-solving ability (for those unable to master the depth of the taught content). Students with improved comprehensive problem-solving ability and mastery of difficult points had a strong cognitive performance rate of 97.9%, compared with 86.2% for those without mastery. Among students without improved innovation ability and without mastery of the depth of the taught content, those with improved comprehensive problem-solving ability had a strong cognitive performance rate of 50.0%, compared with 15.8% for those without improvement (Figure 2).

## Discussion

The COVID-19 pandemic, as a major public health event, has had a profound impact on the development of *Clinical Epidemiology*. This discipline plays a pivotal role in understanding clinical syndromes and addressing critical challenges such as pandemic response (3, 18, 19). In the era of big data, the scope of clinical epidemiology has been enriched and expanded through novel approaches for data acquisition and analysis, as well as the introduction of new theories and methods such as translational research and precision medicine. These advances provide valuable supplementation and extension for the growth and consolidation of the discipline (20–22). In parallel, ongoing educational reforms have prompted universities worldwide to explore innovative teaching models and pedagogical strategies. Among these, the BOPPPS blended teaching model has gained increasing popularity. By integrating participatory learning and flipped classroom components with case analysis and literature sharing, it encourages students to articulate their perspectives actively (10, 23, 24). When students engage fully in classroom discussions and practical activities—particularly when they are able to propose innovative research designs or problem-solving strategies—their cognitive performance tends to be more robust (25, 26).

This study aimed to evaluate the impact of a BOPPPS-based blended teaching model on cognitive performance and to identify key influencing factors. The results support the proposed hypothesis that the BOPPPS hybrid teaching model effectively promotes cognitive performance in *Clinical Epidemiology* instruction. Specifically, the study identified that higher levels of student engagement and the development of higher-order cognitive skills were associated with better cognitive performance. Innovation capability, knowledge integration, and problem-solving skills emerged as the most significant influencing factors.

The ability to summarize and generalize the fundamental methods of a course reflects not only the students’ mastery of knowledge but also the clarity, organization, and logical structure of their thinking. Within the BOPPPS blended teaching framework, through targeted guidance and explanation from instructors, coupled with self-directed and collaborative learning, students can utilize tools such as mind maps to systematically review the knowledge structure of the course. Simultaneously, they can distill key points and essential content, which supports the development of a coherent knowledge system and fosters more structured and logical thinking (10, 27, 28).

*Clinical Epidemiology* is a methodological discipline that applies the principles and methods of epidemiology to address research problems in clinical medicine. It serves as a bridge between clinical medical research and epidemiology. The primary aim of the clinical epidemiology curriculum is to cultivate students’ ability to identify and solve problems and to conduct scientific research, thereby enhancing their research capacity and cognitive skills (29–31). How to foster students’ thinking skills through the dynamic interaction between teaching and learning has long been a focus of active exploration for our instructional team.

With the deepening of institutional teaching reform and the advancement of high-quality course development—coupled with the team’s ongoing pedagogical innovation—our *Clinical Epidemiology* course has adopted a teaching approach that integrates the BOPPPS model with blended learning. The present study found statistically significant differences in scores across various dimensions according to gender, degree type, interest in the chosen discipline, adaptability to campus life, and personality traits. Further analysis using a decision tree revealed that five factors were key determinants of overall cognitive performance: improvement in innovative capacity, ability to summarize and generalize the fundamental methods of the course, ability to master the depth of the knowledge taught, ability to grasp the difficult points of the content, and improvement in the comprehensive ability to solve complex problems.

These findings provide an evidence base for improving future teaching practices. First, in curriculum content design, teaching should remain responsive to current developments, align with the training objectives of different degree programs, and emphasize personalized instruction. Second, sustained emphasis should be placed on cultivating students’ innovation and problem-solving abilities, encouraging them to integrate acquired knowledge with practical applications. This can be achieved by leveraging institutional and departmental platforms to promote active participation in research projects and competitions, thereby enabling students to discover, analyze, and address problems in practice while enhancing their skills. Third, the BOPPPS blended teaching model should be continuously optimized by enriching course resources and refining assessment systems to better meet students’ needs and foster their academic and personal development.

The BOPPPS blended teaching model implemented at our institution fully leverages the advantages of online resources and competition-based learning opportunities. Through the development of virtual learning platforms and participation in academic and technological innovation competitions—such as academic work contests and modeling competitions—students are provided with greater opportunities to gain a deeper understanding of knowledge, internalize it, and apply it in practice. This approach not only enables students to master factual knowledge but also enhances their capacity to explore its depth, thereby fostering the ability to analyze and discuss problems from multiple perspectives rather than limiting themselves to superficial phenomena, ultimately broadening both the depth and breadth of their think (10, 28, 32).

The ability to master challenging knowledge points reflects a high level of cognitive capacity and problem-solving skills. In the teaching process, instructors draw on preliminary assessments of students’ learning needs to employ case-based teaching methods, helping them break down complex and difficult concepts. Additionally, through the sharing of online resources, simulation of real-world clinical problems, and implementation of project-based learning, students are encouraged to actively explore key issues in clinical and public health domains. They are guided to integrate their acquired knowledge and skills to systematically and effectively analyze problems, propose reasonable solutions or research plans, and critically evaluate and refine their own proposed solutions or plans (26, 33).

Compared with traditional teaching models, the BOPPPS blended teaching approach for the *Clinical Epidemiology* course at our institution is underpinned by the philosophy of Outcome-Based Education (OBE), with the entire instructional process being student-centered. This model transforms the classroom from a one-way knowledge transmission to an interactive dialog, from a closed setting to an open learning environment, from a focus solely on content acquisition to one that emphasizes competency development, from prioritizing rote learning over critical thinking to integrating learning with reflective thinking, and from teacher-dominated instruction to a learning-centered paradigm. Through the use of diversified teaching strategies and activity designs, the model stimulates students’ intellectual engagement and places particular emphasis on cultivating their capacity for solving complex problems, fostering innovation, and developing higher-order thinking skills, thereby enhancing their overall cognitive performance (34, 35).

### Limitations

This study has several limitations in terms of participant selection, questionnaire design, and data collection and analysis. For example, the participants were limited to graduate students from our institution who had enrolled in this course, and the questionnaire items were designed using only dichotomous options. Several potential sources of bias should also be considered. First, selection bias may have occurred because participants were from a single institution. Second, information bias may exist due to the use of self-reported questionnaire data. In future work, and when conditions permit, we plan to expand the sample size and conduct comparative studies involving both students who have and have not taken the *Clinical Epidemiology* course. In addition, we will continue to refine the questionnaire design to more comprehensively and accurately capture the outcomes of the survey. Future studies should adopt longitudinal or experimental designs, incorporate multi-center data, and explore advanced analytical models to validate and extend these findings.

## Conclusion

Implementing the BOPPPS blended teaching model in *Clinical Epidemiology* courses exerts a significant positive impact on students’ overall thinking performance. In future teaching practice, this model should be fully valued and appropriately applied to cultivate high-caliber, interdisciplinary medical and health professionals who possess a solid foundation in epidemiological knowledge, demonstrate strong and efficient learning abilities, actively enhance their capacity for knowledge transfer, and are equipped with creative thinking to meet future challenges.

## Data Availability

The original contributions presented in the study are included in the article/Supplementary material, further inquiries can be directed to the corresponding authors.
