# Supplementary material for: The impact of a decision tree–based BOPPPS blended teaching model on cognitive performance in clinical epidemiology
Source: Front Public Health. 2026 May 25;14:1854470. doi: 10.3389/fpubh.2026.1854470 (PMC13243371; doi:10.3389/fpubh.2026.1854470)
Supplement: Supplementary file 1 [file Table_1.docx]

# Evaluation Form for the High-Quality Course Construction of Clinical Epidemiology

Instructions:
This questionnaire aims to understand the effects of the blended teaching model in the Clinical Epidemiology course, so as to provide reference for the development of diversified teaching models in medical universities. Please check (√) the option that best represents your true feelings. Thank you for your support and cooperation.

## Part I. Personal Information

1. Gender [Single choice] *

○ Male ○ Female

2. Age (years) [Fill in the blank] * _________________________________

3. Ethnicity [Single choice] *

○ Han ○ Ethnic minority *

4. Place of household registration [Single choice] *

○ Urban ○ Rural

5. Grade [Single choice] *

○ First-year postgraduate student

6. Degree type [Single choice] *

○ Academic degree ○ Professional degree

7. How much do you like your major? [Single choice] *

○ Like ○ Do not like

8. What is your opinion on the prospects of your major? [Single choice] *

○ Good ○ Not good

9. How well do you adapt to the current campus environment (living, learning, and natural environment)? [Single choice] *

○ Adapt ○ Do not adapt

10. What do you think your personality type is? [Single choice] *

○ Introverted ○ Extroverted ○ Intermediate

## Part II. Knowledge Mastery

11. Ability to clearly define learning objectives [Single choice] *

○ Yes ○ No

12. Ability to explain the basic definitions of the course [Single choice] *

○ Yes ○ No

13. Ability to explain the research scope of the course [Single choice] *

○ Yes ○ No

14. Ability to explain the tasks of the course [Single choice] *

○ Yes ○ No

15. Ability to summarize the basic principles of the course [Single choice] *

○ Yes ○ No

16. Ability to summarize the basic methods of the course [Single choice] *

○ Yes ○ No

17. Ability to grasp the breadth of the knowledge taught [Single choice] *

○ Yes ○ No

18. Ability to grasp the depth of the knowledge taught [Single choice] *

○ Yes ○ No

19. Ability to grasp the key points of the knowledge taught [Single choice] *

○ Yes ○ No

20. Ability to grasp the difficult points of the knowledge taught [Single choice] *

○ Yes ○ No

## Part III. Improvement in Learning Abilities

21. Efficient learning ability [Single choice] *

○ Improved ○ Not improved

22. Knowledge transfer ability [Single choice] *

○ Improved ○ Not improved

23. Autonomous learning ability [Single choice] *

○ Improved ○ Not improved

24. Problem discovery ability [Single choice] *

○ Improved ○ Not improved

25. Problem analysis ability [Single choice] *

○ Improved ○ Not improved

26. Problem-solving ability [Single choice] *

○ Improved ○ Not improved

27. Comprehensive ability to solve complex problems [Single choice] *

○ Improved ○ Not improved

28. Literature search ability [Single choice] *

○ Improved ○ Not improved

29. Literature reading ability [Single choice] *

○ Improved ○ Not improved

30. Clinical research ability [Single choice] *

○ Improved ○ Not improved

31. Innovation ability [Single choice] *

○ Improved ○ Not improved

32. Clinical practice ability [Single choice] *

○ Improved ○ Not improved

33. Language expression ability [Single choice] *

○ Improved ○ Not improved

34. Teamwork and communication ability [Single choice] *

○ Improved ○ Not improved

## Part IV. Development of Thinking

35. Critical thinking [Single choice] *

○ Formed ○ Not formed

36. Innovative thinking [Single choice] *

○ Formed ○ Not formed

37. Epidemiological thinking mode [Single choice] *

○ Formed ○ Not formed

38. Thinking for constructing a knowledge system [Single choice] *

○ Formed ○ Not formed

39. Thinking of knowledge transfer and integration [Single choice] *

○ Formed ○ Not formed

40. Rigorous scientific research thinking [Single choice] *

○ Formed ○ Not formed

## Part V. Teaching Satisfaction

41. Teaching content [Single choice] *

○ Satisfied ○ Not satisfied

42. Teaching method [Single choice] *

○ Satisfied ○ Not satisfied

43. Teaching effect [Single choice] *

○ Satisfied ○ Not satisfied

44. Teaching atmosphere [Single choice] *

○ Satisfied ○ Not satisfied

## Part VI. Overall Evaluation of Teaching Method

45. Difficulty of teaching content [Single choice] *

○ Difficult ○ Not difficult

46. Acceptability of teaching content [Single choice] *

○ Acceptable ○ Not acceptable

47. Acceptability of teaching method [Single choice] *

○ Acceptable ○ Not acceptable

48. Contribution of teaching method to comprehensive quality cultivation [Single choice] *

○ Helpful ○ Not helpful

49. Contribution of teaching method to clinical professional skills cultivation [Single choice] *

○ Helpful ○ Not helpful

50. Contribution of teaching method to further advanced study [Single choice] *

○ Helpful ○ Not helpful
